# Identification of novel therapeutic targets and effective anticancer agents for gallbladder cancer by integrating bioinformatics analysis and experimental validation

**DOI:** 10.3389/fphar.2025.1658462

**Published:** 2025-12-18

**Authors:** Yanjie Zhong, Ruiqi Zou, Ximei Luo, Siqi Yang, Zhiqiang He, Haijie Hu, Fuyu Li

**Affiliations:** 1 Division of Biliary Tract Surgery, Department of General Surgery, West China Hospital, Sichuan University, Chengdu, Sichuan, China; 2 Institute of Fundamental and Frontier Sciences, University of Electronic Science and Technology of China, Chengdu, China; 3 Yangtze Delta Region Institute (Quzhou), University of Electronic Science and Technology of China, Quzhou, Zhejiang, China

**Keywords:** biliary tract tumors, organoid, mendelian randomization, network pharmacology, single-cell transcriptome, molecular docking

## Abstract

**Background:**

Although significant progress has been made in the treatment of biliary tract tumors (BTC), most patients still respond poorly to existing therapies. Therefore, the development of new therapeutic targets and drugs remains an urgent need. Previous studies have extensively applied multi-omics approaches to identify pathogenic targets and drug candidates; however, experimental validation has often been insufficient.

**Methods:**

To identify therapeutic targets associated with biliary tract tumors (BTC), we performed Mendelian randomization (MR) analyses integrating cis-eQTL data of druggable genes with BTC GWAS datasets to determine potential therapeutic targets. Subsequently, drug repurposing analyses were conducted to identify candidate compounds corresponding to these druggable gene targets, which were further validated through molecular docking and experimental verification. Single-cell transcriptomic analysis was used to explore the effects of key targets on the tumor microenvironment and tumor progression.

**Results:**

MR analysis identified eight genes associated with biliary tract tumors. Among them, NT5E and C4B were prioritized as key regulatory nodes through protein–protein interactions (PPI) network analysis. Drug prediction and molecular docking identified myricetin as a candidate molecule targeting NT5E with strong binding affinity, which was subsequently confirmed in cellular and patient-derived organoid (PDO) models. Single-cell transcriptomic analysis revealed that NT5E was predominantly expressed in C4_CD8-CD8A T cells, which exhibited cytotoxic yet immunosuppressive phenotypes, contributing to immune evasion and poor prognosis.

**Conclusion:**

This study identifies potential therapeutic targets for BTC. Drugs designed to target these genes have a higher likelihood of clinical success and are expected to facilitate BTC drug development while reducing associated costs.

## Introduction

Biliary tract tumors (BTC) encompass intrahepatic cholangiocarcinoma, extrahepatic cholangiocarcinoma, gallbladder carcinoma, and ampullary carcinoma (Valle et al., 2021), collectively accounting for approximately 3% of all gastrointestinal tumors (Valle et al., 2021; Benavides et al., 2015; Hundal and Shaffer, 2014). Due to their aggressive and nonspecific characteristic, most BTC are diagnosed at an advanced stage, with only 20% of patients eligible for curative surgery (Vogel et al., 2023; Shroff et al., 2019; Primrose et al., 2019). For the majority with unresectable disease, systemic therapy remains the mainstay. The ABC-02 Phase III trial established the combination of gemcitabine with cisplatin (GC) as the standard first-line chemotherapy for advanced BTC (Turkes et al., 2019). However, the 5-year overall survival rate remains below 5% (Bridgewater et al., 2016), and even post-surgical patients receiving adjuvant capecitabine relapse within a median of 25.9 months (Primrose et al., 2019). The treatment needs of BTC patients are far from met, underscoring the urgent need for new therapeutic approaches.

Recent advances in bioinformatics have enabled the investigation of potential pathogenic targets, enabling targeted interventions for frequently mutated genes like MET, ERBB2 (HER2), FGFRs, and IDH1 (Kendre et al., 2023; Harding et al., 2023; Capuozzo et al., 2022; Kam et al., 2021). Among these approaches, Mendelian Randomization (MR) effectively reduces confounding and reverse causality, making it increasingly adopted for exploring disease etiology. MR explores potential causal relationships between pathogenic genes and tumors by integrating summary expression quantitative trait locus (eQTLs) studies and disease data (Su et al., 2023; Storm et al., 2021; Gaziano et al., 2021). In parallel, online DSigDB databases have been widely utilized to predict candidate drugs for specific therapeutic targets, followed by molecular docking to assess protein-drug interactions and medicinal activity (Chen et al., 2024; Li et al., 2024). To further validate our findings, patient-derived organoids (PDOs), which closely recapitulate the structural and genomic features of primary tumors and better maintain tumor heterogeneity than traditional 2D cultures (Bruun et al., 2020; Wang et al., 2021), are utilized to evaluate the therapeutic efficacy of agents identified through bioinformatic prediction and molecular docking.

In this study, we propose several innovative discoveries in the field of biliary tract tumor research. First of all, we utilize Mendelian randomization method to identify the causal effects of blood druggable eQTLs on BTC. Secondly, gene enrichment analysis reveals the functional characteristics and biological relevance of potential therapeutic targets. We also construct protein–protein interaction network to screen out the critical node NT5E. Additionally, single-cell analysis suggests that NT5E was mainly expressed in cytotoxic and immunosuppressive C4_CD8-CD8A T cells, promoting immune evasion and poor clinical outcomes. Lastly, several Chinese medicines that may have therapeutic effects on biliary tract tumors are selected through drug prediction, molecular docking, organoid and cellular experiments. Our experiments reveal that myricetin treatment reduces the proliferation and migration abilities of GBC cells, accompanied by the downregulation of NT5E expression.

## Materials and methods

### Data acquisition, specimen collection and cell culture

**FIGURE 1:**
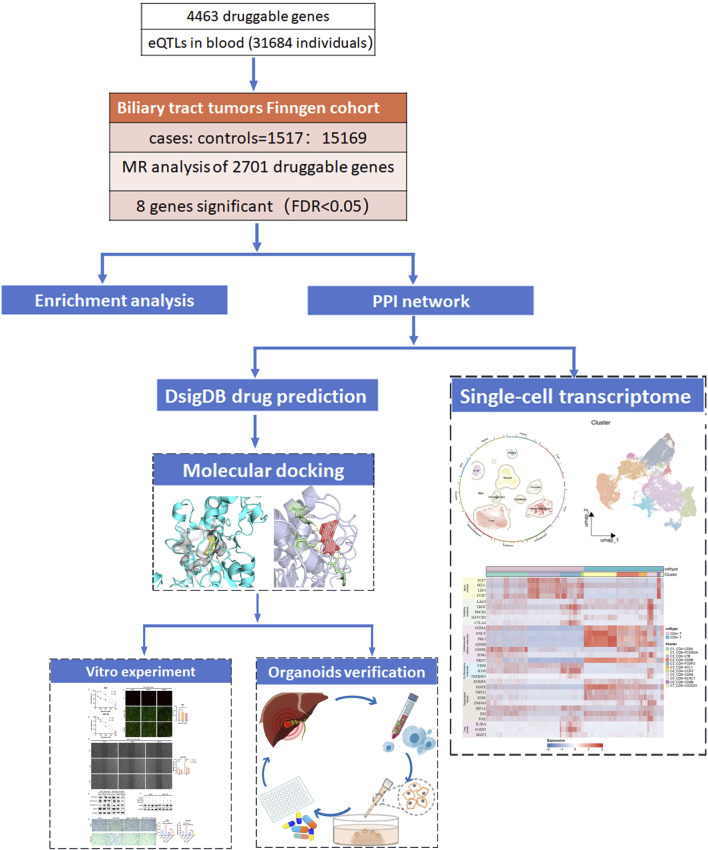
Workflow of the study. A series of analyses was conducted to identify druggable genes associated with Biliary tract tumors (BTC). 4463 druggable genes were extracted from the recent published review (Finan et al., 2017). Integration of Finngen summaries and cis-eQTLs data from the blood by using Mendelian randomization methods prioritized blood druggable genes associated with the risk of BTC (FDR <0.05). Functional enrichment analysis was conducted on the initially selected genes, and subsequent PPI network analysis further narrowed the range of key genes. On one hand, single-cell data were used to validate the role of the selected genes in the tumor microenvironment. On the other hand, effective small molecule drugs were predicted based on MsigDB database, and their efficacy was evaluated through molecular docking and experimental verification.

A flowchart of the study is shown in Figure 1. This MR study utilized blood eQTL data from eQTLGen Consortium (https://eqtlgen.org/) and GWAS data from the FinnGen consortium (ID: finngen_R10_C3_BILIARY_GALLBLADDER_EXALLC, https://www.fnngen.f/en) to assess the potential causal relationships between genes and cancer. Additionally, 4,463 druggable genes linked to known drugs were obtained from a recent study and are listed in Supplementary Table S1 (Finan et al., 2017). Finally, the single cell transcriptome sequencing dataset GSE201425 was obtained from GEO.

### Mendelian randomisation analysis

The Mendelian randomization (MR) analyses were performed using the R package TwoSampleMR (V.0.5.6) (Hemani et al., 2018). The eQTLs of the druggable genes were selected as the exposure data. The instrumental variables (IVs) used for MR analysis were subject to the following conditions: (1) SNPs with FDR<0.05 were identified as directly associated with exposure; (2) SNPs were not associated with exposure-outcome confounders; (3) SNP solely influences the outcome through the exposure. Furthermore, instrumental variables with F-statistics (F=(beta/se)2) greater than 10 were retained to reduce bias caused by weak instrumental factors. Finally, we remove linked SNPs at r2<0.2 and a cropping range of 1,000 Kb using a reference profile from the 1,000 Genome Project (Abec et al., 2012), retaining the most significant SNPs. The outcome data were then loaded and harmonised using the “harmonize_data” function. In the primary analysis, MR estimates were calculated for each SNP using the Wald ratio method. In cases where multiple SNPs exist, inverse variance weighted (IVW), MR-Egger and weighted median methods were chosen for further analysis. Horizontal pleiotropy can be assessed through MR-Egger intercept test. On the flip side, we next assessed heterogeneity using Cochran’s Q, determined by both the IVW and MR-Egger methods. The corrected FDR value served as the significance threshold, where an FDR of <0.05 was deemed statistically significant.

### Network pharmacology research

To comprehend the biological function of the promising pharmaceutical targetable genes derived from the aforementioned MR Analysis, we conducted Gene Ontology (GO) and Kyoto Encyclopedia of Genes and Genomes (KEGG) analyses. To unravel the intricate intracellular interactions among the proteins encoded by the aforementioned candidate genes, we imported the targets into the STRING database for Protein-Protein Interaction (PPI) analysis and subsequently visualized using Cytoscape 3.7.2.

### Drug prediction and molecular docking

Using the Drug Signatures Database (DSigDB, http://dsigdb.tanlab.org/DSigD Bv1.0/) on the Enrichr platform to predict small molecule drugs targeting key nodes of PPI network (Yoo et al., 2015). Uploading the identified target genes to DSigDB also allows us to obtain medicinal evaluation metrics such as the combined score or probability of each drug’s interaction with the target gene.

We totally downloaded NT5E (4H2I) and C4B(6YSQ) from the PDB library (Sehnal et al., 2021) (https://www.rcsb.org/). Small molecules structure was downloaded from PubChem database (Kim et al., 2021) (https://pubchem.ncbi.nlm.nih.gov/) and the corresponding IDs have been displayed in Figure 3A. AutoDock Vina (V1.2.2) were used to dewater, hydrogenate (Eberhardt et al., 2021). Grid boxes were strategically positioned to encompass the structural domains of each protein, permitting free molecular movement. Finally, visualizing the structure of interaction of small molecule ligand drugs and receptor proteins with the PyMOL software.

### Histology and staining

Specimens were fixed with 10% neutral buffered formalin (Sigma–Aldrich) at room temperature for 24 h. The paraffin embedding was performed as follows: samples went through a graded ethanol series, xylene and then paraffin. The embedded samples were cut into 5 μm and prepared for immunohistological (IHC) staining according to a standard protocol. For IHC, anti-CD73 Rabbit pAb (Servicebio, 1:500) were used.

### Establishment of organoids

GBC samples were obtained from patients who underwent radical resection in the Department of Biliary Surgery, West China Hospital of Sichuan University. All patients’ diagnoses were histologically confirmed. The specimen is stored in preservation solution at 4° before being transported to the laboratory (within 12 h).

Patient-derived specimens were minced and incubated in digestive fluid (2.5 mg/mL Collagenase Type II, Gibco) with a single-cell suspension preparation apparatus for 30–40 min. And then add the TrypLE Express (Gibco, 12,605-010) to the water bath for digestion for 1 min, filter with a cell filter (100 μm), and centrifuged at 1,000 rpm for 5 min. The pellet was washed three times using Dulbecco’s modified eagle medium (DMEM) with 1% fetal bovine serum (FBS), 1% penicillin/streptomycin, 1% hydroxyethyl piperazine ethanesulfonic acid (HEPES) and 1% Glutamax, and centrifuge at 1,000 rpm for 5 min.

The remaining cells mixed with Matrigel matrix (Corning) were seeded and cultured in forty-eight-multiwell suspension plate. The organoid culture medium for normal and GBA samples consisted of Advanced DMEM/F12 medium (Gibco), 1% Glutamax, 1% HEPES, 1% penicillin/streptomycin, 1.25 mM N-acetyl-l-cysteine (Sigma), 1:50 B27 supplement (Gibco), 1:100 N2 supplement (Gibco), 1:500 Primocin (Invitrogen), 25 ng/mL recombinant human HGF, 100 ng/mL FGF10 (Peprotech), 100 ng/mL FGF2 (Peprotech), 100 ng/mL recombinant human Noggin (Peprotech), 50 ng/mL EGF (Peprotech), 10 nM gastrin (Sigma), Wnt3a, 10 mM Y27632, 5 μM A8301, 10 μM forskolin (Tocris Bioscience), 10 mM nicotinamide, 5 μg/mL Prostaglandin E2 and recombinant human R-Spondin1.

### Drug treatment

Briefly, dissociated cancer cells were resuspended in 2 µL Matrigel matrix mixture and then seeded onto the 98-well plate (100 µL complete culture medium per well). The cells were allowed to recover at 37 °C for 4 days and 100 µL of complete culture medium containing 10 µM of chrysin, myricetin, luteolin, quercetin and acacetin was added into the wells. Dimethyl sulfoxide (DMSO) was used as blank control. After the treatment, the morphologic changes of organoids were recorded by photographing every 24 h.

### Cell culture and cell transfection

Human GBC-SD and NOZ were acquired from Shanghai Key Laboratory of Biliary Disease Research. The cells were maintained in DMEM containing 10% fetal bovine serum, and incubated at 37 °C in a humidified atmosphere of 5% CO2.

All plasmids were products of GeneChem (Shanghai, China). The shRNA sequences were listed in Supplementary Table S2. Cells were seeded in a 6-well plate and grown up to 70% confluence after 24 h. And then plasmids were transfected using Lipofectamine 3000 Transfection Reagent (Invitrogen, United States), according to the manufacturer’s instructions. The cells were harvested after transfection for 24 h to perform follow-up assays.

### Real-time reverse transcription-quantitative polymerase chain reaction (RT-qPCR)

Total RNAs was extracted using a FastPure Cell/Tissue Total RNA Isolation kit (Vazyme, China) following the manufacturer’s instructions. Total RNA was reverse-transcribed using HiScript III (Vazyme), followed by qPCR analysis with SYBR Green Master Mix (Vazyme) on a Bio-Rad CFX96™ system. The primer sequences used for RT-qPCR are listed in Supplementary Table S3.

#### Cell proliferation assay

GBC cells were seeded into 96-well plates at 4000 cells per well and cultured overnight at 37 °C. After 24/48 h treated with or without myricetin, the cytotoxicity was assessed by incubating the cells with CCK-8 reagent (BS350B, Biosharp) for 1–2 h, and measuring the absorbance at 450 nm. The EdU (5-Ethynyl-2′-Deoxyuridine) proliferation assay was determined by using EdU detection kits (KeyGEN BioTECH, China) according to the manufacturer’s instruction. 10 μM EdU solution were added into cells for 2 h, immobilized with 4% polyoxymethylene for 30 min, and permeabilized with 0.5% Triton-X100. Cells were stained with 1× Click-iT EdU kFluor555 solution for 30 min at room temperature in the dark, and then stained with 1 × Hoechst 33,342. Cells were detected by the fluorescence microscope and analyzed by ImageJ.

### Transwell assay

Cells (3 × 10^4^) in serum-free DMEM (200 μL) were seeded in Matrigel-uncoated Transwell inserts. After 24 h incubation (37 °C, 5% CO_2_), migrated cells on the lower membrane were fixed (4% PFA, 40 min), stained (1% crystal violet), and quantified (5 fields/insert) using ImageJ (v2021.8.0). Data represent three replicates.

### Western blot

Proteins collected from cells were resolved by 10% sodium dodecyl-sulfate polyacrylamide gel electrophoresis (SDS-PAGE) and then transferred onto polyvinylidene difluoride (PVDF) membrane (Merck, Germany). These membranes were blocked with 5% nonfat dry milk for 1 h at room temperature and incubated with primary antibodies overnight at 4 °C. After three washes with TBST, the membranes were incubated with secondary antibodies at 25 °C for 1 h. After repeated washing, the results were visualized using a chemiluminescence imager with abundant supersensitive ECL luminescent solution.

### Single-cell transcriptome analysis

For scRNA‐seq analysis, the primary tool used was the ‘Seurat’ R package (version 4.0.2). Cells meeting the following criteria were chosen for future analysis: (1) filtering out genes expressed in fewer than 5 cells and cells detecting fewer than 300 distinct genes; (2) 200 < nFeature_RNA < 5000; (2) cells with mitochondrial transcript levels exceeding 15%.

### Dimensionality reduction, clustering, and annotation

After exclusion of low-quality cells, UMI counts were normalized by the function ‘NormalizeData’. Thereafter, ‘FindVariableGenes’ function was performed with default parameters to detect 2000 highly variable genes. The datasets collected from different samples were integrated using the RunHarmony function to mitigate batch effects. The data were scaled using ‘ScaleData’ and the first 30 principle components were adopted for auto-clustering analyses using ‘FindNeighbors’ and ‘FindClusters’ functions. For all 75,215 cells, we identified nine cell types with the resolution parameter as 0.1. The 36,993 T cells were further categorized into 11 clusters with a resolution parameter of 0.2.

#### Pathway RespOnsive GENes algorithm

For single-cell data, we inferred oncogenic signaling pathway activity scores using the human version of PROGENy (version 1.22.0) based on the top 500 most responsive genes (Schubert et al., 2018; Ho et al., 2020). The original pathway activity scores were inferred based on the t-values of gene sets provided by the PROGENy package for the targeted pathways.

### Cell-cell communication

We utilized the “CellChat” R package (v1.6.1) to conduct intercellular interaction analysis, which could predict the probability of intercellular communication as well as the associated pathways.

### Definition of naive, inhibitory, cytokines and other scores of different T cell subtypes

We considered published functional analyses of cytotoxicity and effects of different T cell subtypes (Zheng et al., 2020; Navarro-Barriuso et al., 2018). Naive, inhibitory, cytotoxic and effectiveness, Co-Stimulatory, Transcription and Treg scores were evaluated by combining expression of gene sets with different functional properties in each T cell subpopulation (Liu et al., 2022).

## Results

### Identification the vital druggable gene based on Mendelian randomization analysis

**FIGURE 2:**
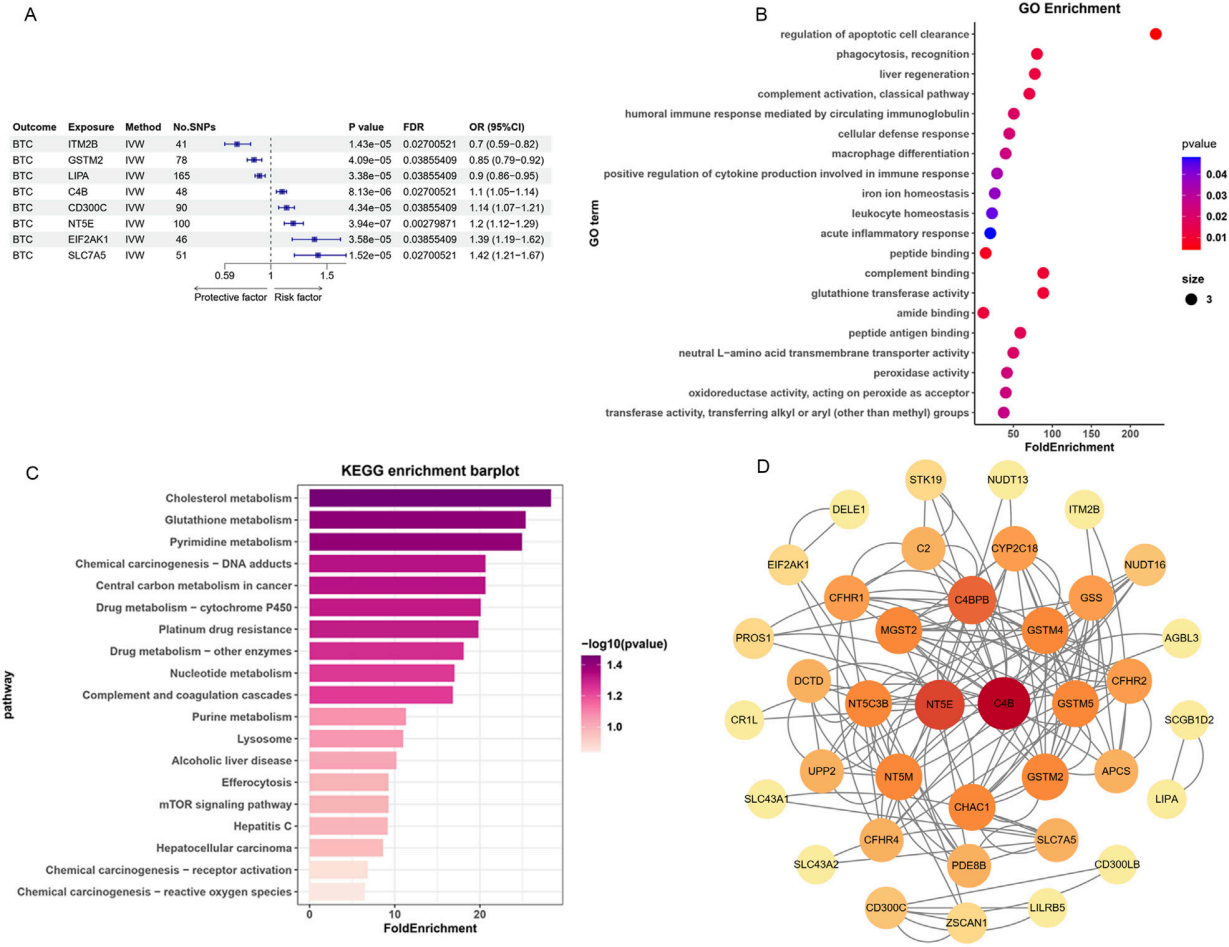
The identification and functional Characteristics of potentially druggable genes. **(A)** Forest plots displaying the findings from the Mendelian randomization analysis phase for 8 significant genes. **(B,C)** Bar plots showing the top enriched GO **(B)** and KEGG **(C)** pathway terms. **(D)** PPI network built with STRING.

By integrating allele frequency statistics, we filtered cis-eQTLs with a false discovery rate (FDR) of less than 0.05. After intersecting with the eQTLs data, we identified eQTLs associated with 2554 druggable genes. Mendelian randomization analysis was then conducted to investigate the causal relationship between genes and biliary tract cancers (BTC), revealing that 8 genes are significantly associated with tumor risk (FDR<0.05, Figure 2A). When sensitivity analyses were performed, all the gene had consistent direction of effect values across the three methods (Supplementary Table S4), but the LIPA genes failed the horizontal pleiotropy test (P < 0.05, Supplementary Table S5), so these three genes were excluded from subsequent analyses. Additionally, the heterogeneity test results (Supplementary Table S6) demonstrated no heterogeneity among the SNPs of candidate genes.

### Enrichment analysis

GO and KEGG functional enrichment analysis were performed to further explore the physiological functions of these aforementioned hub genes and their potential links with diseases. The enrichment of GO entries suggested that these genes might be involved in the regulation of apoptotic cell clearance, phagocytosis, complement activation, humoral immune response mediated by circulating immunoglobulin and other immune-related processes (Figure 2B). Moreover, the enriched KEGG pathways predominantly centered around metabolic processes, encompassing Cholesterol, Glutathione, and Central carbon metabolism in cancer. Furthermore, pathways like the mTOR signaling transduction pathway, platinum drug resistance, and drug metabolism-related enzymes were also enriched. These findings suggest the involvement of multiple targets and pathways in the intricate processes of biliary tumor occurrence, development, and treatment (Figure 2C).

### Key targets on PPI network

To elaborate the interaction among the putative targets, we constructed a protein interaction network utilizing the STRING database and visualized it using Cytoscape software. The final 8 target genes PPI network consisted of 38 nodes and 70 edges (Figure 2D). The C4B and NT5E in the networks were the highest degree of value, followed by C4BPB (7), CHAC1(6) and GSTM2(6) (Supplementary Table S7). Therefore, we hypothesize that C4B and NT5E serve as pivotal nodes in the protein interaction network and may represent the most promising candidate genes for drug targeting.

### Candidate drug prediction

**FIGURE 3:**
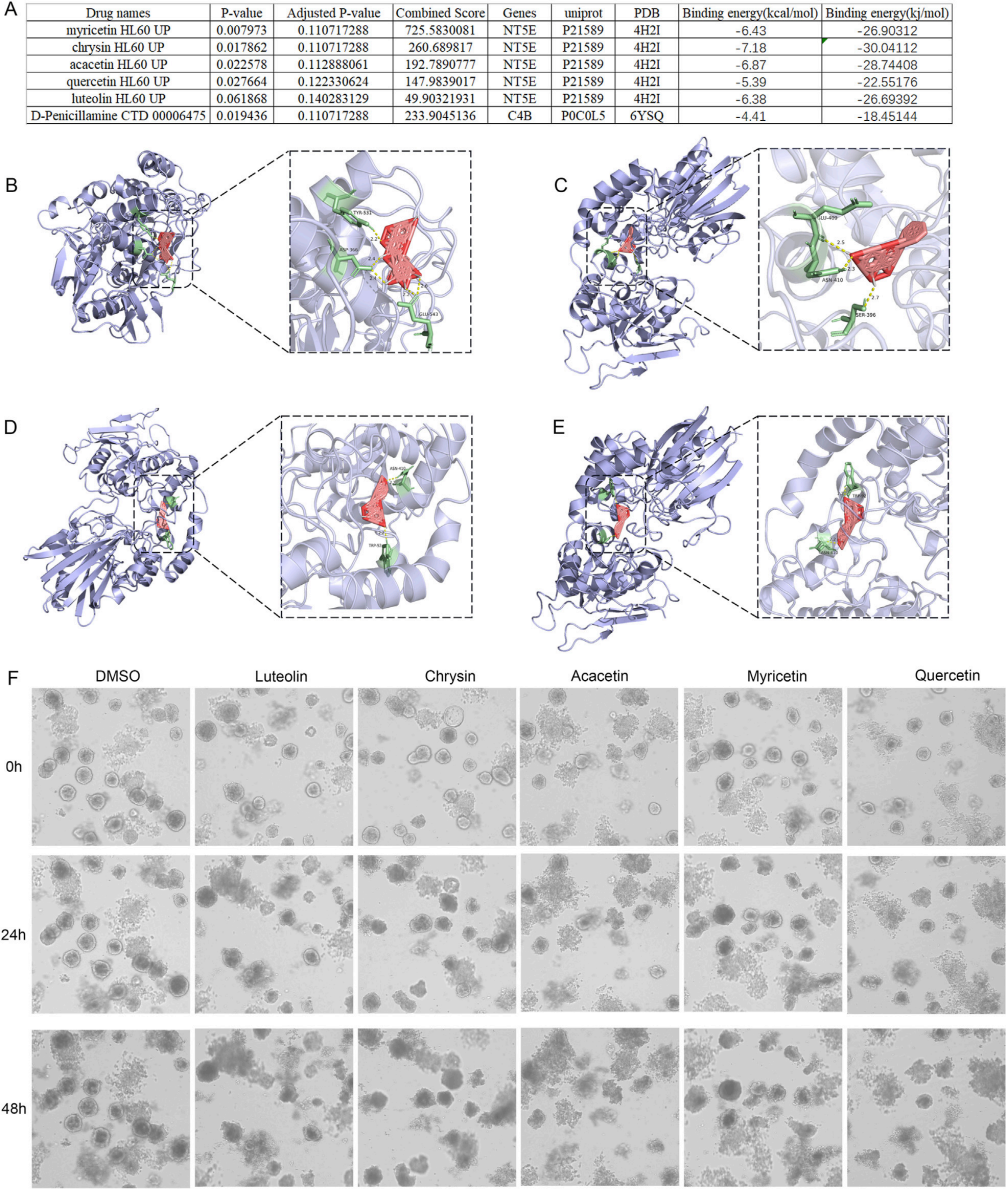
Predicting small molecule drugs targeting potentially druggable genes. **(A)** Docking results of available proteins with small molecules. **(B)** Docking results of NT5E and myricetin. **(C)** Docking results of NT5E and chrysin. **(D)** Docking results of NT5E and acacetin. **(E)** Docking results of NT5E and quercetin. **(F)** Representative brightfield microscopy images of GBC organoids after treatment with DMSO and 10uM luteolin, chrysin, acacetin, myricetin, and quercetin every 24 h.

In this study, we utilized the DSigDB database available on the Enrichr platform to predict potential intervention drugs that could be effective to BTC. The top drugs were selected for further analysis based on the combination scores of target genes and drugs (Figure 3A). The results showed that myricetin (myricetin HL60 UP), chrysin (chrysin HL60 UP), acacetin (acacetin HL60 UP), quercetin (quercetin HL60 UP) and luteolin (luteolin HL60 UP) were the top five potential chemical compounds, which targeted to NT5E.

### Molecule docking and organoid identification

Leveraging the DSigDB database from the Enrichr website, we searched for potentially effective drugs targeting the key genes NT5E and C4B. Myricetin has the highest combined score, making it the most promising drug molecule for targeting NT5E in the treatment of BTC. Traditional Chinese medicines like myricetin, chrysin, acacetin, quercetin, and luteolin showed binding energies below −5 kcal/mol (Figure 3A), suggesting their potential as NT5E-targeting therapies for biliary tract tumors (Zhu et al., 2023). The docking results were visualized through PyMOL (Figures 3B–E). Additionally, our study results indicate that after 24 h of treatment with 10 µM myricetin and chrysin, GBC PDOs exhibited growth inhibition and morphological changes. (Figure 3F).

### Elevated NT5E expression correlates with poor prognosis in gallbladder carcinoma

**FIGURE 4:**
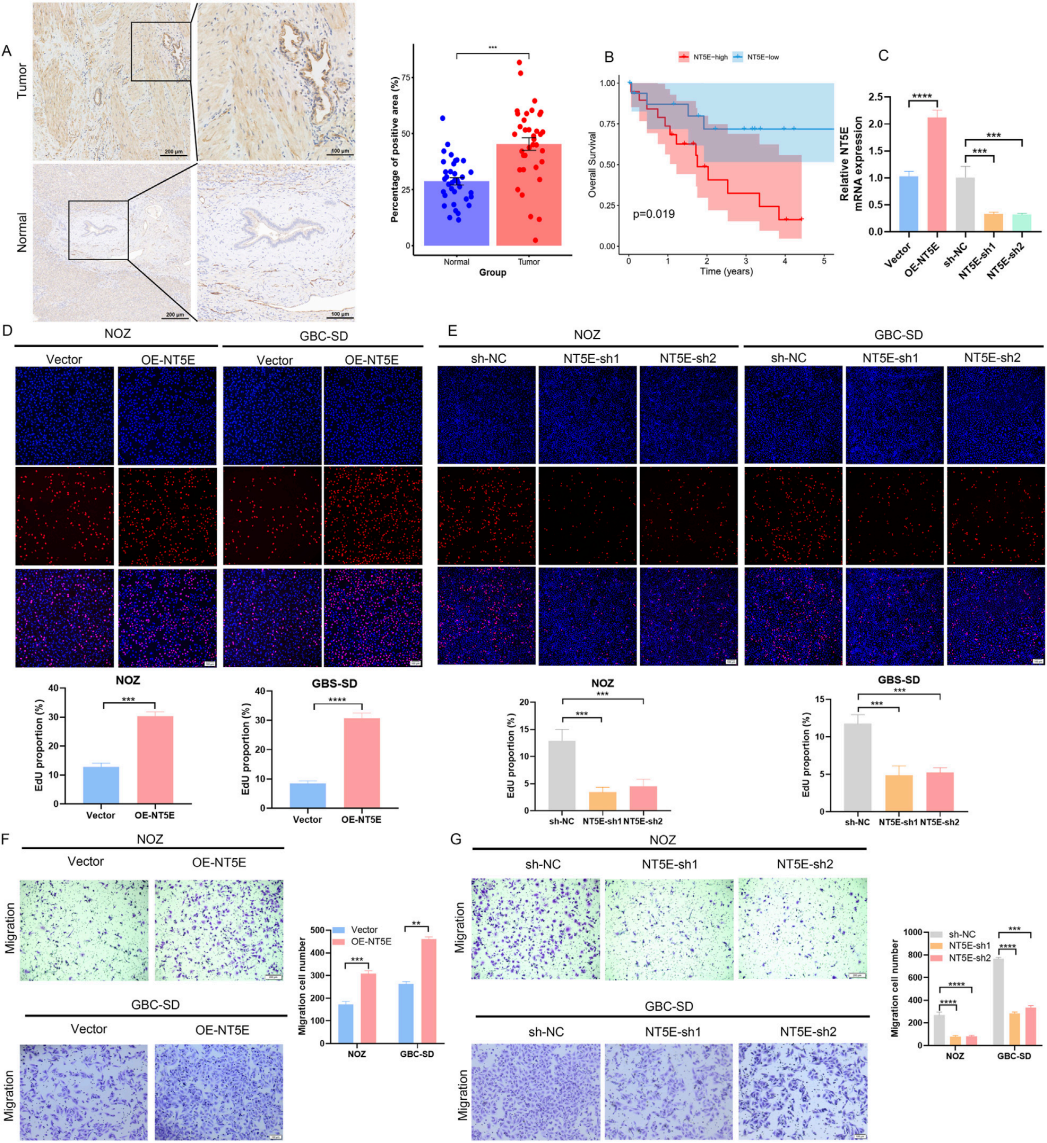
High expression of NT5E in GBC associated with poor prognosis and promotes tumor proliferation and migration. **(A)** IHC method detected NT5E protein expression pattern in GBC tumor and normal adjacent tissues. **(B)** Kaplan-Meier analysis on the correlation of NT5E expression level with GBC patients’ overall survival. **(C)** qRT-PCR tests on NT5E mRNA in shRNA and overexpressed plasmid transfected GBC cell. **(D,E)** The EdU assay was carried out to analyze proliferation abilities of GBC cells with overexpression **(D)** or knockdown **(E)** of NT5E (n = 3). **(F,G)** Representative images of Transwell assays to detect the migration of NOZ (scale bar: 200 μm) and GBC-SD (scale bar: 100 μm) cells with NT5E overexpression **(F)** or knockdown **(G)** (n = 3). Data were assessed with unpaired Student’s t-test or one-way ANOVA and presented as mean ± SD. **P < 0.01; ***P < 0.001.

In this research, a total of 36 matched GBC tumor and adjacent normal tissue pairs were used for protein level detection. IHC assay showed that NT5E elevation in GBC tumor tissues (Figure 4A). Subsequently, patients were divided into NT5E-high and -low expression groups according to the median NT5E expression level. Kaplan-Meier analysis revealed that NT5E-high expression group had significantly shorter overall survival (OS) (Figure 4B). To further investigate the function of NT5E in GBC, NT5E-specific shRNAs and overexpression vectors were transfected and validated in NOZ and GBC-SD cells (Figure 4C). The EdU assays showed NT5E dramatically increased the proliferative cell proportion (Figures 4D,E). Transwell assays revealed that overexpressing NT5E enhanced the migration abilities of GBC cells (Figure 4F), whereas NT5E knockdown exhibited the opposite effects (Figure 4G).

### Myricetin suppresses GBC cell growth by down-regulating NT5E expression

**FIGURE 5:**
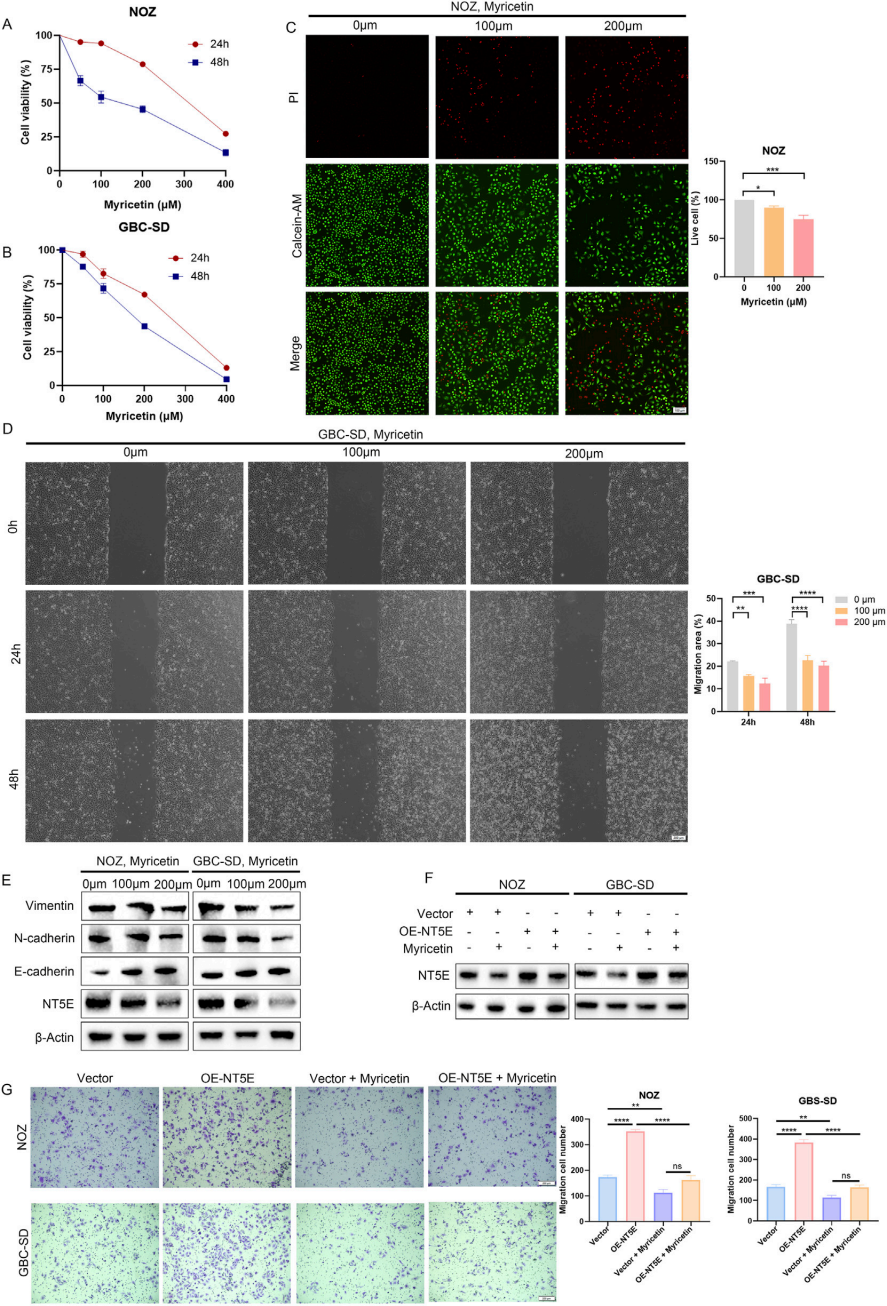
The effects of myricetin on the proliferation and migration of GBC cells. **(A,B)** Effects of myricetin on proliferation of NOZ and GBC-SD cells at 24 h and 48 h. **(C)** Calcein-AM/PI confirmed the death level of myricetin treated NOZ cells. **(D)** Migration capacity of myricetin treated NOZ cells. **(E)** EMT biomarkers (E-cadherin, N-cadherin, Vimentin) and NT5E expression in NOZ and GBC-SD cells treated with myricetin for 24 h detected by Western blot. **(F)** GBC cells transfected with vector or NT5E plasmid were treated with 100 μM myricetin for 24 h, NT5E expression was detected by Western blot. **(G)** Transwell assays to detect the migration ability of NT5E overexpressing/control GBC cells with/without 100 μM myricetin treatment. Data were assessed with unpaired Student’s t-test or one-way ANOVA and presented as mean ± SD. n = 3. *P < 0.05; **P < 0.01; ***P < 0.001; ns, no significance.

GBC cells treated with myricetin were incubated with the CCK-8 reagent. Results showed that the viability of GBC cells treated with myricetin for 24/48 h was obviously declined in a dose-dependent manner (Figures 5A,B). Consistently, the Calcein-AM/PI live-dead assay confirmed myricetin’s dose-responsive cytotoxic effects (Figure 5C). Wound healing assays revealed myricetin significantly reduced migratory capacity of GBC cells (Figure 5D). Western blotting confirmed a significant decrease in NT5E expression following myricetin treatment for 24 h (Figure 5E). However, the overexpression of NT5E partially saved NT5E downregulation induced by myricetin (Figure 5F). Similarly, we found that NT5E overexpression could partially restore impaired migration capacity caused by 100 µM myricetin pretreatment for 24 h (Figure 5G).

### Revealing the role of NT5E in the biliary tract tumor microenvironment using single-cell datasets

**FIGURE 6:**
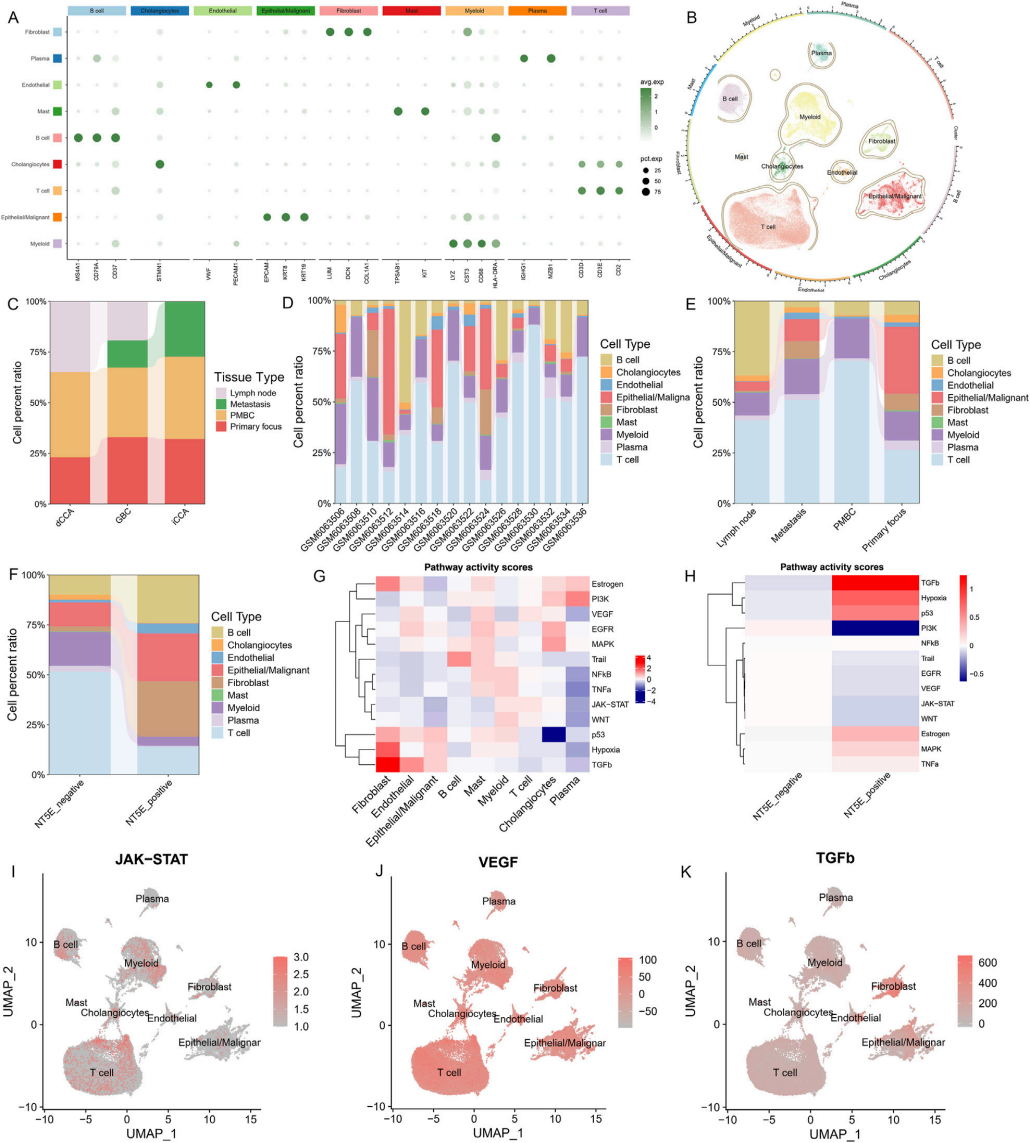
Identifying key cell types in the tumor microenvironment of biliary tract tumors. **(A)** Average gene expression of selected marker genes for cell clusters, dot size indicates the proportion of expressing cells, colored by standardized expression levels. **(B)** Clustering projection according to Seurat’s clustering system using UMAP as the dimension reduction method. **(C–E)** Stacked chart of the proportions of different cell types within the different tumor **(C)**, sample **(D)** and tissue **(E)** sources. **(F)** Differences in cell infiltration between NT5E positive and negative groups. **(G)** Mean pathway activity scores of different cell clusters; **(H)**. The activity scores of common tumor-associated pathways differ between NT5E-positive and NT5E-negative groups. **(I–K)** UMAP display the activity scores of representative pathways across main cell types.

Following stringent filtering, we retained a total of 75,215 cells and 25,817 genes from 16 samples for subsequent analysis. By referring to the specific marker genes of each cluster, we summarized these clusters into 7 cell types (Figures 6A,B). The distribution of cells in tissue types showed great heterogeneity (Figures 6C,D). Notably, primary tumor and metastatic tissue characterized by a high proportion of epithelial/malignant cell, while less T cell infiltrated (Figure 6E). Similarly, NT5E-positive group also exhibit reduced T cell inflitration (Figure 6F). Enrichment analysis revealed that the NT5E-positive group exhibited higher activity in most cancer-related pathways, with considerable intercellular heterogeneity in pathway activation patterns (Figures 6G,H). Figure 6I and J depict representative pathways that are evidently active in T cells, and TGFb was particularly enriched in fibroblasts (Figure 6K).

### Ligand-receptor interaction analysis confirms enhancement of crosstalk in NT5E-positive group

**FIGURE 7:**
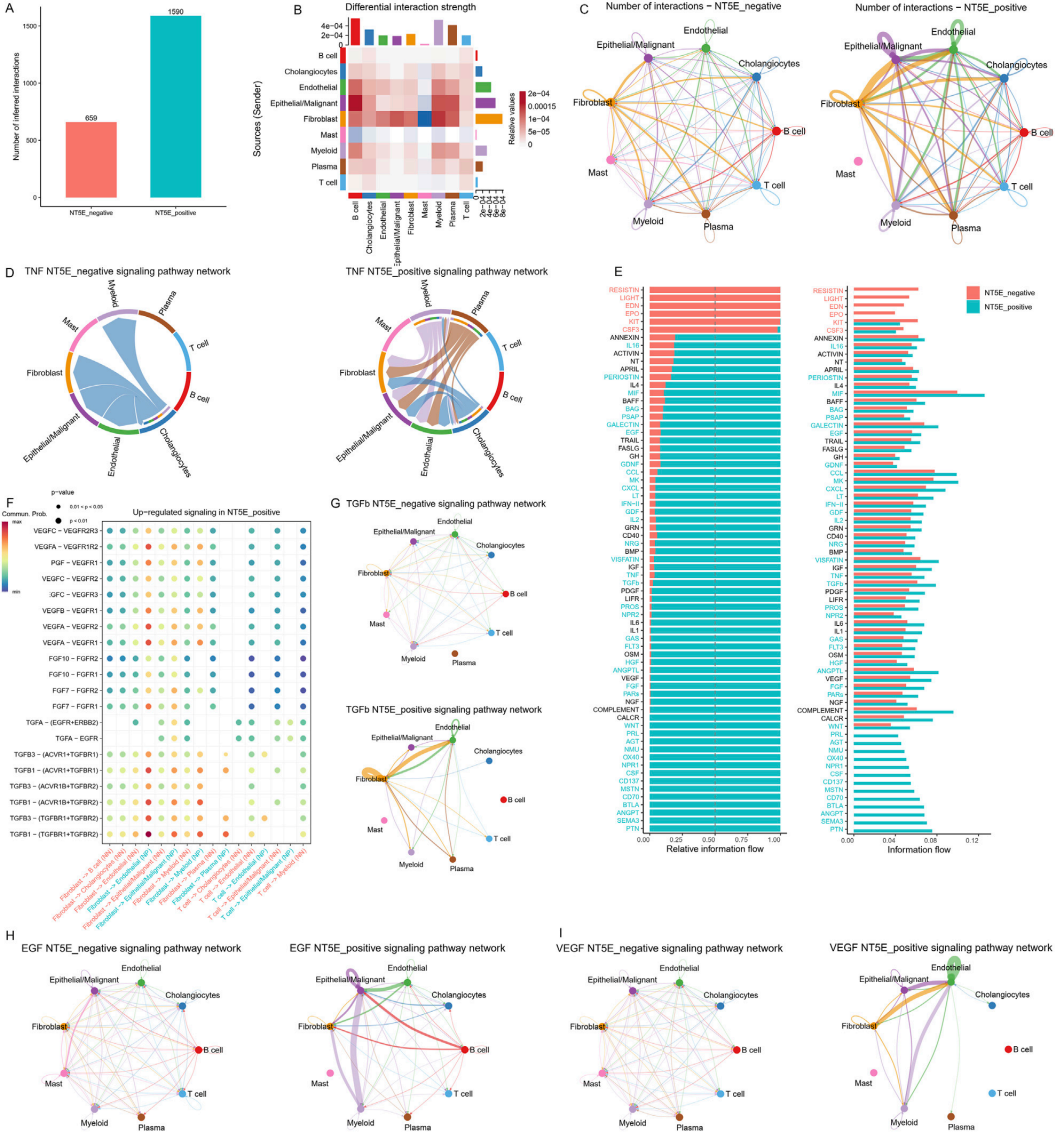
Analysis of intercellular communication among various cell types. **(A)** Bar graph illustrating the quantity of cell communication between the NT5E-positive and -negative groups. **(B)** Heatmap illustrating the strength of cell communication between the NT5E-positive and -negative groups. **(C)** Chord plot of the quantity of cell communication among different cell types in the NT5E -positive and -negative groups. **(D)** Network plots for inferred TNF ligand-receptor interaction activity split by different NT5E groups, depicting the direction of cell communication indicated by arrows. **(E)** Inferred number of Ligand-Receptor (LR) Interactions with cell clusters stratified by different NT5E groups. **(F)** Communication probabilities of important ligand-receptor pairs different between NT5E-positive and -negative groups, with the dot color reflecting the communication probability, blank indicating the communication probability zero, and dot size representing the p value. **(G–I)** Differences in signal transmission of representative signaling pathways (the TGFb pathway, the EGF pathway and the VEGF pathway) between different cell populations in NT5E groups.

CellChat analysis was performed to uncover the intricate intercellular networks. Overall, the NT5E-positive group exhibited more frequent intercellular communication (Figures 7A,B). More specifically, fibroblasts, endothelial and epithelial/malignant cells in the NT5E-positive group showed stronger and more frequent communication with other cells compared to those in the NT5E-negative group (Figure 7C). Circular plots of TNF pathway network confirmed enhanced signals from the myeloid and plasma cells, and decreased signals from the cholangiocytes in NT5E-positive group (Figure 7D).

Differential analysis of intercellular information flow between NT5E-positive and negative groups, key signaling pathways were ranked. Notably, pro-tumor pathways including TNF, EGF, VEGF, FGF, and WNT are enriched in the NT5E-positive group (Figure 7E). It was observed that fibroblasts more frequently interact with endothelial and epithelial/malignant cells in NT5E-positive group, predominantly via the TGFB1-(TGFBR1+ TGFBR2), VEGFA-VEGFR1 ligand–receptor pairs (Figure 7F). In addition, enhanced TGFb and VEGF signaling pathways were found to occur mainly between fibroblasts and endothelial cells (Figures 7G,H). The enhanced VEGF, EGF signaling pathway mainly occurs between tumor cells and endothelial cells (Figure 7I).

### Clustering and subtype analyses of T cells

**FIGURE 8:**
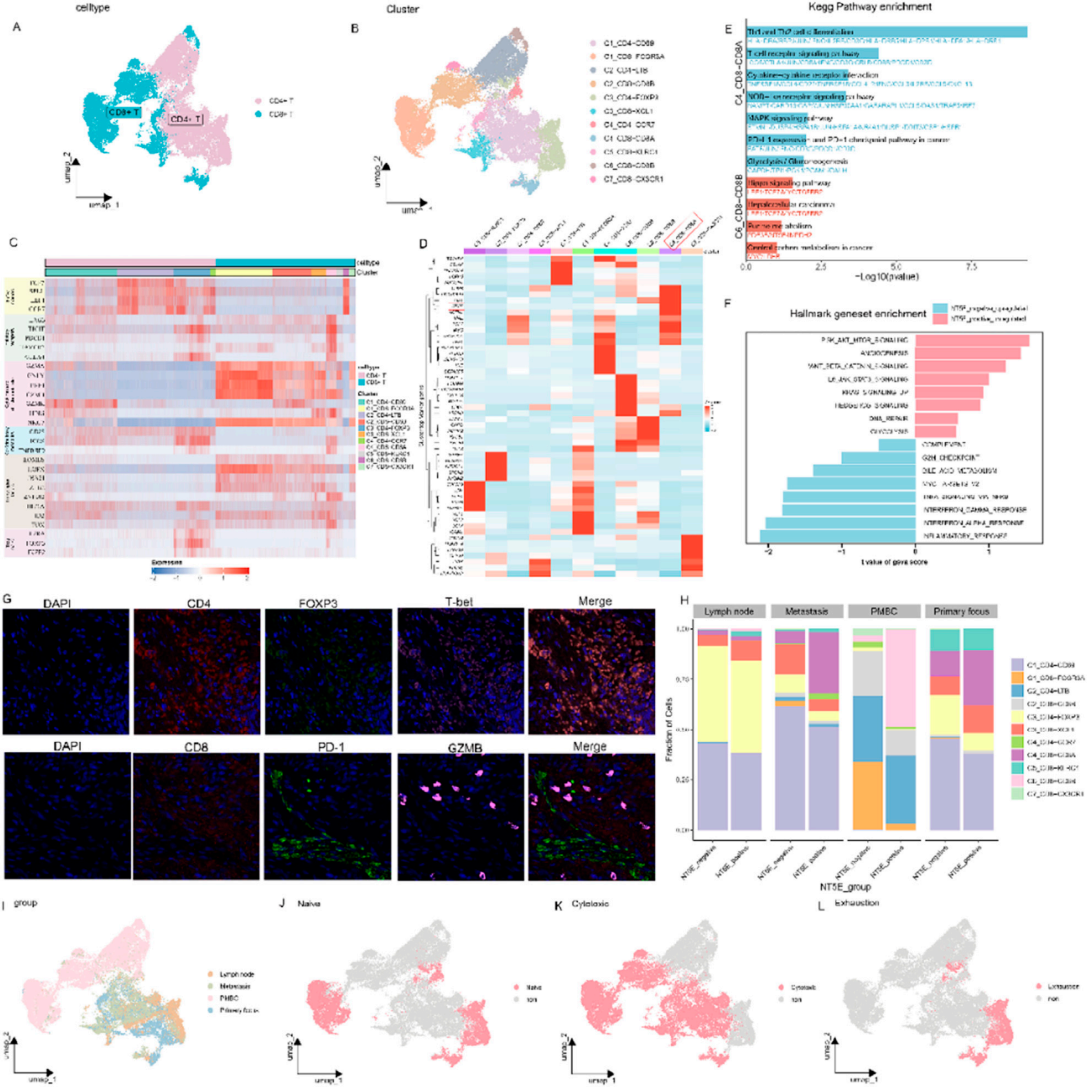
The distribution and functional characteristics of T cell subpopulation. **(A)** The UMAP view of CD4 and CD8 T cell clusters. **(B)** UMAP view of 11 T cell clusters. **(C)** Heatmap of genes associated with naïve, inhibitory, cytotoxicity, co-stimulatory, transcription and treg function across T cell subsets in scRNA seq data. **(D)** Differentially expressed genes (DEGs) of 11 T cell clusters between NT5E-positive and -negative group. **(E,F)** KEGG and Hallmark geneset enrichment analysis of above DEGs. **(G)** Multiplex immunofluorescence images demonstrating the representative expression of Treg and inhibitory marker gene in CD4 and CD8 cells. CD4 and CD8 (red), FOXP3 (Treg marker, green), T-bet (pink), PD-1 (inhibitory marker, green), GZMB (cytotoxicity and effector marker, pink). **(H)** Differences in the distribution of T cell subsets in different tissue sources and NT5E groups. **(I–L)** UMAP view of cell density **(I)** and T cell states **(J–L)** demonstrating main T cell subgroups distribution across four tissue groups.

As the major cytotoxic immune cells in tumors, T cells showed distinct compositional patterns between NT5E groups, necessitating unsupervised clustering (Figures 8A,B). Using established functional markers, we classified T cell into distinct phenotypes: naïve, inhibitory, cytokine and effector, co-stimulatory, transcription and Tregs (Figure 8C). The top differentially expressed genes of each cluster are shown in Figure 8D. NT5E was found to be highly expressed primarily in the C4_CD8-CD8A cell population, which exhibited high cytotoxic and inhibitory scores. KEGG enrichment analysis using marker genes showed that C4_CD8-CD8A were mainly enriched into immune-related pathways such as Th1 and Th2 cell differentiation, cytokine receptor interaction, as well as tumor-related pathways such as MAPK and PD-L1 (Figure 8E). GSVA analysis also found that C4_CD8-CD8A cells in the NT5E positive group were mainly enriched in pro-tumor pathways, such as PI3K_AKT_MTOR, angiogenesis, wnt_beta_catenin signal pathway (Figure 8F). Immunofluorescence staining of gallbladder carcinoma tissue reveals that CD4 cells highly express the Treg cell marker, while CD8 cells show high expression of the inhibitory molecule PD-1 and the cytotoxic molecule GZMB (Figure 8G). Cytotoxic and naïve T cell were less prevalent in NT5E-positive lymph nodes, primary, and metastatic sites, but enriched exhausted/inhibited C4_CD8-CD8A populations (Figures 8H,I). Consistent with previous findings, normal peripheral blood contained abundant naïve and cytotoxic T cells but virtually no exhausted subsets, while lymph nodes and primary lesions were predominantly enriched with exhausted T cells (Figures 8J–L).

## Discussion

In this study, based on blood druggable eQTLs, we identified eight druggable gene that may influence BTC outcomes, including ITM2B, GSTM2, LIPA, C4B, CD300C, NT5E, EIF2AK1 and SLC7A5. This finding was validated by 3 MR methods, including IVW, MR-Egger and weighted median method. Horizontal multiplicity test and Cochran’s Q heterogeneity test were performed to exclude pleiotropy and heterogeneity, and finally all genes except LIPA passed the test. In addition, PPI network was constructed to find the crucial nodes in the disease protein interaction network to further narrow the range of druggable targets. Subsequent single-cell analysis identified NT5E as a key regulator of the tumor microenvironment that promotes cancer progression. Based on these findings, we predicted, molecularly docked, and experimentally validated potential NT5E-targeting drugs.

Ecto-5′-nucleotidase (NT5E/CD73) is crucial in cancer and immune regulation. It converts AMP to adenosine, promoting tumor growth, migration, and suppressing antitumor immune responses (Allard et al., 2014; Loi et al., 2013). NT5E is also involved in epithelial-mesenchymal transition (EMT) induced by TGF-β1, predicting poor outcomes in hepatobiliopancreatic tumors (Xiong et al., 2014; Sciarra et al., 2019). Subsequently, single-cell data was divided into NT5E-positive and -negative groups based on its expression to unveil its role in the biliary tract tumor microenvironment. NT5E-positive cells exhibited enhanced TGFβ pathway activation, which plays a crucial role in the metastasis cascade of gallbladder cancer (Wu et al., 2024). Intercellular communication analysis also indicated enhanced signaling of pro-tumor TNF, EGF, VEGF, FGF, and WNT among cells in the NT5E-positive group. Furthermore, NT5E was predominantly highly expressed in the C4_CD8-CD8A cell group, which exhibited cytotoxic and inhibitory phenotype.

During drug prediction, several traditional Chinese medicine (TCM) were screened with significant p-values and high binding affinities to targetable genes. TCM are widely employed in China as maintenance treatments following conventional cancer treatments like surgery or chemotherapy (Wang et al., 2018; Zh et al., 2020). Among the five screened TCM compounds, myricetin and quercetin have demonstrated anti-tumor efficacy in biliary tract cancers through multiple mechanisms (Tuponchai et al., 2019; Zhao et al., 2019), whereas the remaining three have not been previously reported. We also found that myricetin and chrysin showed more pronounced growth inhibition and morphological changes in gallbladder cancer organoids compared to others. Previous studies demonstrated that myricetin exert anti-tumor activity through modulation of key cancer hallmarks (Li et al., 2019). Our study demonstrates that myricetin downregulated critical oncogenic gene NT5E in gallbladder cancer cells, consequently inducing proliferation inhibition.

Our study also has some limitations. Firstly, the Finngen database were mostly restricted to European populations, which might restrict the applicability of our findings to other groups and races. However, the eQTLs analysis involved individuals of non-European descent, which might lead to biases in MR effect estimates due to genetic background differences. Secondly, because the outcome data is the integrated data of BTC, and the proportion of cholangiocarcinoma and gallbladder cancer is not clear, the prediction of the therapeutic effect of drugs on specific cancers still needs further exploration and analysis. Thirdly, the anti-cancer effects of candidate drugs were primarily evaluated through basic molecular assays and organoid models. Further mechanism studies and clinical trials are necessary to gain a more comprehensive understanding.

## Conclusion

In conclusion, this study utilized Mendelian randomization (MR) analysis to identify potential therapeutic targets for biliary tract tumors (BTC), highlighting NT5E and C4B as key nodes within the protein-protein interaction (PPI) network. By integrating drug prediction, molecular docking, and experimental validation, myricetin was found to suppress BTC progression by downregulating NT5E expression. Future research will focus on elucidating the precise mechanisms involved and conducting preclinical studies to further evaluate these findings.

## Data Availability

Publicly available datasets were analyzed in this study. This data can be found here: Blood eQTL data was downloaded from eQTLGen Consortium (https://eqtlgen.org/) and GWAS data was downloaded from the FinnGen consortium (https://www.fnngen.f/en). Additionally, 4,463 druggable genes linked to known drugs were obtained from a recent study (Finan et al., 2017) and are listed in Supplementary Table S1. Finally, the single cell transcriptome sequencing dataset GSE201425 obtained from GEO.

## Abbreviations

BTC, biliary tract tumors; MR, Mendelian randomization; KEGG, Kyoto Encyclopedia of Genes and Genomes; GSVA, gene set variation analysis; ECM, extracellular matrix; TCM, Traditional Chinese Medicine; eQTLs, expression quantitative trait locus; PDOs, patient-derived organoids.
